# Process Evaluation of Implementing a Pharmacist-Led Intervention to Improve Adherence to Antihypertensive Drugs Among Patients with Type 2 Diabetes in Indonesian Community Health Centers

**DOI:** 10.3389/fphar.2021.652018

**Published:** 2021-05-05

**Authors:** Sofa D. Alfian, Job F. M. van Boven, Aulia Iskandarsyah, Rizky Abdulah, Eelko Hak, Petra Denig

**Affiliations:** ^1^ Unit Pharmaco-Therapy, -Epidemiology and -Economics, Groningen Research Institute of Pharmacy, University of Groningen, Groningen, Netherlands; ^2^ Department of Pharmacology and Clinical Pharmacy, Faculty of Pharmacy, Universitas Padjadjaran, Jatinangor, Indonesia; ^3^ Centre of Excellence in Higher Education for Pharmaceutical Care Innovation, Universitas Padjadjaran, Jatinangor, Indonesia; ^4^ Department of Clinical Pharmacy and Pharmacology, University Medical Centre Groningen, University of Groningen, Groningen, Netherlands; ^5^ Medication Adherence Expertise Centre of the Northern Netherlands (MAECON), Groningen, Netherlands; ^6^ Department of Clinical Psychology, Faculty of Psychology, Universitas Padjadjaran, Jatinangor, Indonesia

**Keywords:** medication adherence, counselling, community pharmacists, process evaluation, RE-AIM framework, Indonesia, low-and middle income countries, LMIC

## Abstract

**Introduction:** A pharmacist-led intervention in Community Health Centers (CHCs) in Indonesia targeted at patients with type 2 diabetes non-adherent to antihypertensive drugs resulted in a significant improvement in adherence to these drugs. The aim of this study was to evaluate the process of implementation this intervention intended to improve adherence to antihypertensive drugs from both the pharmacist and the patient perspective.

**Methods:** Using the RE-AIM (Reach, Effectiveness, Adoption, Implementation and Maintenance) framework, we conducted a focus group among pharmacists (N = 5) and a survey among patients with complete follow-up (N = 44) participating in the intervention group.

**Results:** All pharmacists adopted the provided training and found support tools useful. The pharmacists implemented the intervention as intended (adequate intervention fidelity >69%). Factors relevant for implementation included having sufficient time and confidence, home visits for specific patients, multidisciplinary collaboration, and availability of a personal counseling room. To maintain the intervention, the need for practical guidance and support from health care authorities was mentioned. Most patients (96%) were satisfied with the information provided by the pharmacists and they believed the tailored counselling was helpful. Most patients (84%) reported that the duration of counselling was sufficient. The large majority of patients would like to receive the counselling regularly.

**Conclusion:** Positive effects of the pharmacist-led intervention can be explained by adequate levels of reach, adoption and implementation in the participating CHCs. For successful implementation and maintenance in Indonesia or other low-and middle-income countries, sufficient training, resources, multidisciplinary collaboration, guidance and support from health care authorities are expected to be important.

## Introduction

Many interventions that have been developed for improving adherence to medication are evaluated in randomized controlled trial (RCT) settings focusing mostly on their clinical effectiveness (Nieuwlaat et al., 2014; Kini and Ho, 2018). Additionally, to inform scale-up of an intervention in real-world daily practice, insight into the implementation of interventions is critically important to understand what caused an intervention to succeed or fail (Carroll et al., 2007). However, an evaluation of the implementation process of adherence management interventions is not always provided (Glasgow et al., 2001; Breitenstein et al., 2010), especially in low and middle-income countries (LMIC) (Correia et al., 2019).

In 2019, we conducted a low-cost pharmacist-led intervention in Community Health Centers (CHCs) in Indonesia targeted at patients with type 2 diabetes non-adherent to antihypertensive medication (Alfian et al., 2021). This intervention resulted in a significant improvement in self-reported medication adherence to these antihypertensive drugs, but no significant changes in secondary outcomes, such as beliefs about antihypertensive drugs, were observed (Alfian et al., 2021). We aimed to evaluate the process of the implementation of this intervention intended to improve adherence to antihypertensive drugs using a structured evaluation.

Several evaluation frameworks have been developed to facilitate the translation of research findings (Tabak et al., 2012; Milat and Li, 2017). The RE-AIM framework is one of the most frequently used evaluation frameworks (Milat and Li, 2017) in a variety of settings and across a broad range of behavioral outcomes (Glasgow et al., 2019). The RE-AIM framework has been developed to evaluate public health interventions assessing five dimensions—Reach, Effectiveness, Adoption, Implementation and Maintenance—at multiple levels (e.g., individual or organization) (Glasgow et al., 1999; Glasgow et al., 2019). We will use the RE-AIM framework to evaluate the implementation process of the intervention from both the pharmacist and the patient perspective.

## Methods

### Study Design, Population and Setting

The process evaluation was part of a previously published cluster RCT performed in Bandung City, Indonesia (Alfian et al., 2020; Alfian et al., 2021). We applied a mixed-methods study design. All pharmacists from CHCs that were approached for the RCT participated in the study. Patients with type 2 diabetes using antihypertensives, aged at least 18 years old, who reported non-adherence to their antihypertensive drugs according to Medication Adherence Report Scale (MARS) and who were randomized to the pharmacist-led intervention group were eligible for the process evaluation. In addition, the pharmacists from the five CHCs that conducted the intervention were included in the process evaluation. The study design, the intervention and its effectiveness have been described in more detail elsewhere (Alfian et al., 2020; Alfian et al., 2021). The study was approved by the Health Research Ethics Committee of Universitas Padjadjaran No. 859/UN6.KEP/EC/2019 and was registered at clinicaltrials.gov under the identifier NCT04023734.

### Summary of the Intervention

In short, the key aspects of the intervention included a 3-hours training on medication adherence management and practical support tools for pharmacists to help identifying patient-specific barriers for medication non-adherence, and providing personalized adherence support strategies (Alfian et al., 2020; Alfian et al., 2021). The adherence support strategies included: (1) reminders/habit-based strategies and involvement of family members to address forgetfulness, (2) counselling to increase knowledge, (3) counselling to increase motivation, and (4) counselling to address other drug-related problems. The support tools included simple flow-charts based on the patients’ responses to the MARS and additional questions derived from the Brief Medication Questionnaire, an adherence intervention wheel, and a personalized patient leaflet containing a self-management plan (Alfian et al., 2020). All patients randomized to the intervention group received pharmacist counselling based on their individual barrier(s) to adherence at baseline and at 1-month follow-up sessions.

### Process Evaluation of the Intervention

The implementation process of the intervention was evaluated using the RE-AIM framework. The RE-AIM framework addresses both internal validity (Effectiveness, Implementation, and Maintenance) and external validity (Reach and Adoption) (Glasgow et al., 2019).

Reach was assessed by measuring the participation rates of patients. Participation rates were measured during the RCT and reasons for non-participation were recorded. Effectiveness has been reported separately, focusing on effects on medication adherence, patients’ beliefs about medication, and blood pressure levels (Alfian et al., 2021). Adoption was evaluated by exploring pharmacists’ and patients’ satisfaction with, and willingness to use various parts of the intervention based on the focus group discussion and survey responses. Implementation was evaluated by determining whether the intervention was delivered as intended (i.e., intervention fidelity), and by exploring pharmacists’ and patients’ suggestions for future implementation based on the focus group discussion and survey responses. Maintenance was assessed by determining whether and how the intervention could be maintained according to the pharmacists. Furthermore, the willingness of pharmacists to continue the intervention as part of routine clinical practice and the willingness of patients to receive adherence counselling at every visit or less often were assessed.

### Data Collection

During and after completion of the RCT, data were collected to evaluate the implementation of the intervention. From the pharmacists, qualitative data were obtained by means of a focus group discussion with all five participating pharmacists. This discussion, led by one of the researchers (SDA), was held after all five pharmacists had delivered the second intervention at the 1-month follow-up session. Given the dynamic nature of the focus groups discussion, participating pharmacists had the opportunity to support opinions that had already been proposed by others, challenge these opinions, suggest new personal opinions, or discuss any disagreements regarding the implementations of the intervention. The outline and probing questions for the focus group discussion are presented in Supplementary Table S1 of the Supplementary data. From the patients, quantitative data were collected three months after their baseline session by means of a survey ( Supplementary Table S2 of the Supplementary data), based on a previously developed survey (Stuurman-Bieze et al., 2013). The survey was administered when the patients visited the CHC by trained research assistants.

Intervention fidelity, that is, the degree to which the intervention is implemented as intended, was assessed after each patient visit using a checklist, which contained the four adherence support strategies. Pharmacists indicated to what extent each adherence support strategy was implemented on a 5-point Likert scale, where 0 = “not at all,” 1 = “minimally,” 2 = “to some extent,” 3 = “a good deal,” and 4 = “a great extent” (Supplementary Table S3 of the Supplementary data). Adequate intervention fidelity was defined as achieving at least “a good deal” for the adherence support strategies that needed to be delivered given the identified barrier(s).

### Data Analysis

The focus group discussion with the pharmacists was audio-taped, transcribed verbatim and analyzed by two researchers (SA, AI) using qualitative analysis software NVivo 12. Any disagreement was discussed between the two researchers until consensus was achieved. The statements in the transcripts were summarized and clustered using the thematic analysis approach based on the dimensions of the RE-AIM framework. The quantitative data from the patient survey were tabulated or presented in a figure as frequencies. Only the results of patients with complete follow-up were reported.

## Results

**FIGURE 1:**
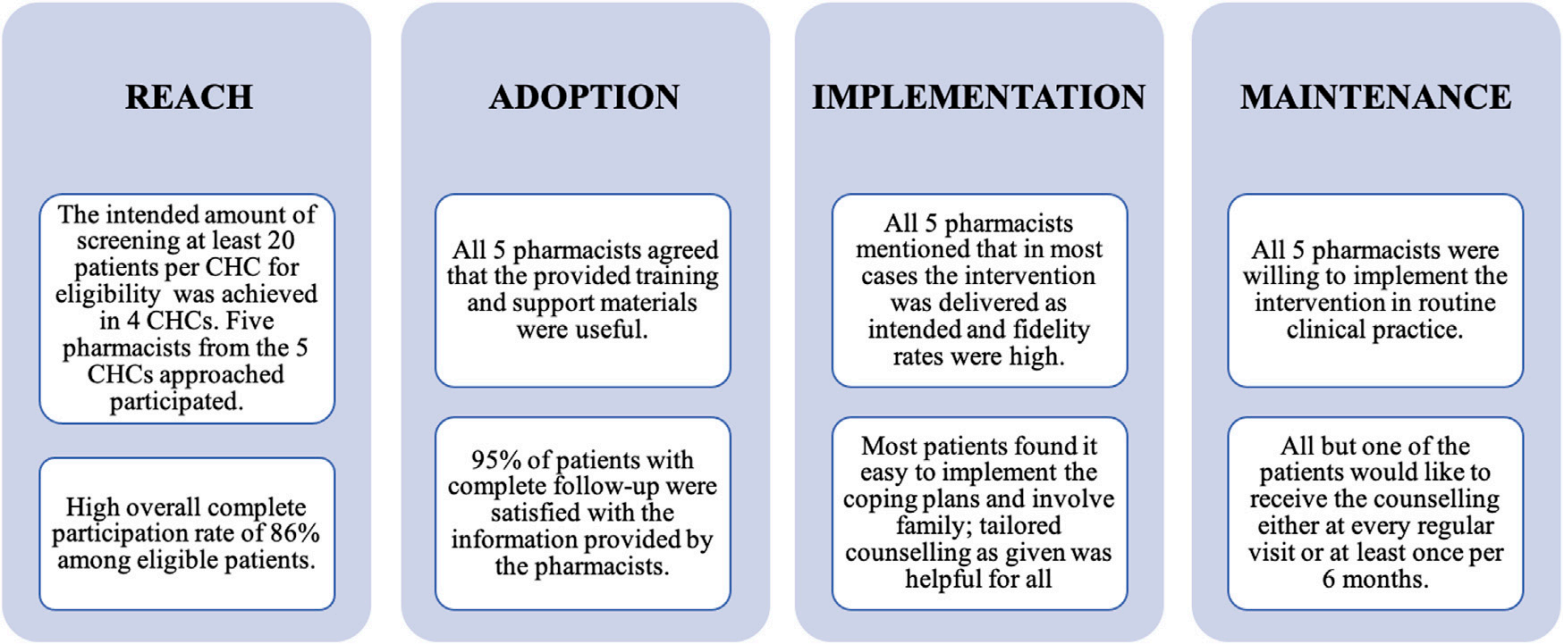
Reach, Adoption, Implementation, and Maintenance dimensions of the RE-AIM* framework from pharmacists’ and patients’ perspectives: main findings. Note: *: Effectiveness has been reported in more detail elsewhere (Alfian et al., 2021).

In total, five pharmacists reporting on 57 enrolled intervention patients contributed to the process evaluation. From these patients, 49 participated in both intervention sessions and 44 out of these 49 completed the process evaluation questionnaire. Overall, most patients were female, aged between 60 and 69 years, and more than a quarter had a low level of formal education, regardless of having complete follow-up in our study (Table 1). The results of the Reach, Adoption, Implementation, and Maintenance dimensions of the RE-AIM framework are summarized in Figure 1.

**TABLE 1:** Baseline sociodemographic characteristics of patients.

Characteristic	Patients with only baseline session (N = 8)	Patients with two intervention sessions (N = 49)	Patients with complete follow-up included in the process evaluation (N = 44)
Gender			
Male	1 (12.5)	13 (26.5)	12 (27.3)
Female	7 (87.5)	36 (73.5)	32 (72.7)
Age (years)			
≤ 49	1 (12.5)	2 (4.1)	2 (4.5)
50–59	3 (37.5)	11 (22.4)	10 (22.7)
60–69	4 (50.0)	25 (51.0)	21 (47.7)
≥ 70	—	11 (22.4)	11 (25.0)
Type of insurance			
BPJS-Non PBI	3 (37.5)	20 (40.8)	15 (34.1)
BPJS-PBI	4 (50.0)	28 (57.1)	28 (63.6)
Missing	1 (12.5)	1 (2.0)	1 (2.3)
Education			
No formal education/elementary school	2 (25.0)	17 (34.7)	13 (29.5)
Junior high school	2 (25.0)	11 (22.4)	10 (22.7)
Senior high school	2 (25.0)	15 (30.6)	15 (34.1)
University	1 (12.5)	6 (12.2)	6 (13.6)
Missing	1 (12.5)	—	—

BPJS-PBI, insurance premium was paid by the government; BPJS-Non PBI, insurance premium was paid by the patients themselves.

### Reach

The intended amount of screening of at least 20 patients per CHC for eligibility was achieved in four out of the five CHCs (80%); in one CHC 17 patients were screened; resulting in a total of 111 patients screened. A high participation rate of more than 95% among patients for the first counselling session was observed. Patient reasons to decline participation were “having no time” and “not feeling well.” This screening resulted in identifying 57 eligible patients for the first counselling session. Forty-nine patients participated in the follow-up session, resulting in an overall complete participation rate among eligible patients of 86%.

### Adoption

All five pharmacists reported that the 3-hours training was useful and clear. After the training, they were willing to use all parts of the intervention in diabetes patients receiving antihypertensive drugs. Regarding the conduct of the intervention, the pharmacists mentioned that they were able to identify patient-specific barriers to non-adherence and that they were easily guided to the corresponding adherence strategies using the support tools. These tools were reported to be clear, easy to use, and informative. All pharmacists agreed that their counselling of patients had become more focused.

The majority of the 44 patients with complete follow-up were (very) satisfied (42; 96%) with the information provided by the pharmacists (Table 2). Most of them agreed that the information provided was (very) important (41; 93%). Moreover, they assessed the atmosphere during the counselling as being (very) relaxed (44; 96%) (Table 2).

**TABLE 2:** Satisfaction with the intervention in patients with complete follow-up (N = 44).

Satisfaction with the information provided by the pharmacist	**Very satisfied**	**Satisfied**	**Neutral**	**Very unsatisfied**	**Unsatisfied**
		12 (27)	30 (68)	2 (5)	0	0
Importance of information provided by the pharmacist	**Very important**	**Important**	**Neutral**	**Unimportant**	**Very unimportant**
		16 (36)	25 (57)	2 (5)	1 (2)	0
How often patients want the counselling when it would be implemented in the future	**At every visit**	**Once per 6 months**	**Once per year**	**Once per 2 years**	**Never**
		24 (55)	19 (43)	1 (2)	0	0
Information provided by the pharmacist was clear	**Totally agree**	**Agree**		**Disagree**	**Totally disagree**
		14 (32)	28 (64)		2 (5)	0
Sufficient opportunity to discuss own experiences and problems with medication	**Totally agree**	**Agree**		**Disagree**	**Totally disagree**
		7 (16)	31 (71)		6 (14)	0
The pharmacist listened well to own experiences and problems with medication	**Totally agree**	**Agree**		**Disagree**	**Totally disagree**
		7 (16)	33 (75)		4 (9)	0
General atmosphere during the sessions	**Very relaxed**	**Relaxed**		**Tensed**	**Very tensed**
		2 (5)	42 (96)		0	0
Number of sessions		**Too less**	**Sufficient**	**Too much**	
		4 (9)	36 (82)	4 (9)	
Time between sessions (1 month)		**Too short**	**Sufficient**	**Too long**	
		7 (16)	37 (84)	0	
Duration of sessions		**Too short**	**Sufficient**	**Too long**	
		3 (7)	37 (84)	4 (9)	

### Implementation

During the focus group discussion, all pharmacists mentioned that in most cases the intervention was delivered as intended. According to pharmacists, most of patients were responsive during the counselling and truly willing to address the identified problems, while only a small number of patients seemed not very interested in making changes.

**FIGURE 2:**
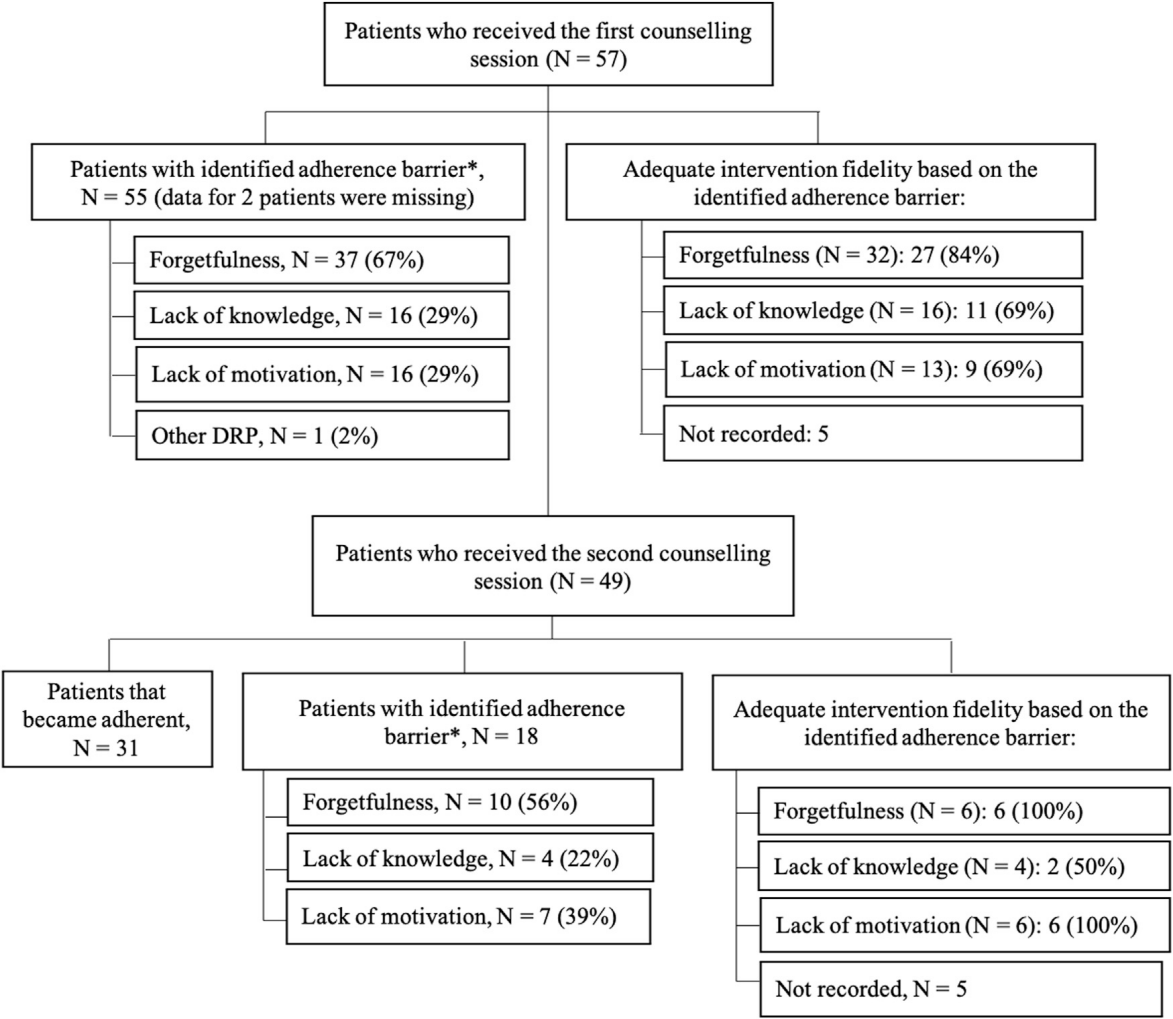
Flow chart of patients with identified adherence barriers and its intervention fidelity. Note: *Patients may have two or more barriers for adherence.

Out of the 57 patients who received the first adherence counseling session, 41 (72%) patients had one barrier for adherence identified and 14 (25%) patients had two or more barriers identified. The most common barrier was forgetfulness, followed by lack of knowledge and lack of motivation (Figure 2). Barrier(s) from two patients were not recorded due to management problems of the pharmacist. Reasons identified for adherence barriers are summarized in Table 3. Intervention fidelity for the first session was high (84%) for strategies addressing forgetfulness, and slightly lower (69%) for strategies addressing lack of knowledge or lack of motivation (Figure 2). Intervention fidelity for five patients was not recorded due to management problems of the pharmacist.

**TABLE 3:** Reasons for non-adherence.

Forgetfulness	Lack of knowledge	Lack of motivation	Other identified drug related problem
1. Patient was busy.	1. Patient did not know the purpose of antihypertensive drugs, either for the short- or long-term.	1. Patient had concerns about side effects of antihypertensive drugs.	Patient had difficulty to refill antihypertensive drugs at the CHC.
2. Patient forgot to take the drug when traveling (for example, when visiting his/her children in another city).	2. Patient only took antihypertensive drugs when he/she had symptoms.	2. Patient did not feel better when taking antihypertensive drugs.	
	3. Patient only took antihypertensive drugs when he/she knew that their blood pressure was high.	3. Patient did not perceive that antihypertensive drugs were safe and preferred to use traditional medicine.	
	4. Patient did not know the importance of taking antihypertensive drugs daily.	4. Patient worried that taking several drugs at the same time would lead to renal problems, especially when other patients or family members already had a renal disease.	
	5. Patient did not know how to deal with possible side effects of antihypertensive drugs.	5. Patient felt bored to take antihypertensive drugs daily.

Among the 49 patients that participated in a 1-month follow-up session, 31 (63%) patients had become adherent according to their MARS scores. Particularly, problems with forgetfulness and lack of knowledge were reduced to a great extent (Figure 2). For the 18 patients who still reported non-adherence, forgetfulness and lack of motivation remained the most common barriers (Figure 2). Adequate intervention fidelity for the required adherence support strategies was high at follow-up session of counselling (Figure 2). Intervention fidelity for five patients was not recorded due to management problems of the pharmacist.

All pharmacists agreed that a follow-up session is necessary to evaluate the patient’s implementation of the agreed recommendation strategies. All pharmacists mentioned that the 3-hours training and the support tools facilitated the implementation of the intervention. Two pharmacists mentioned difficulties to manage time during counselling as well as a lack of confidence and lack of experience with patient counselling. For future implementation, three pharmacists suggested they would prefer to have their own counselling room in order to conduct counselling more comfortably. In three CHCs, the pharmacists shared the counselling room with others health care providers (nutritionist/nurse). Furthermore, two pharmacists indicated that the personalized patient leaflet was rather large, and suggested it should be more concise for practical reasons. Conducting home visits was suggested by three pharmacists in order to monitor medication adherence more objectively, especially for patients at risk of medication related problems. On the other hand, all pharmacists mentioned that the feasibility of conducting home visits is uncertain. Furthermore, one pharmacist mentioned that the pharmacist-led intervention should not be a stand-alone approach but rather a collaboration with other health care providers to optimize pharmaceutical care.

**FIGURE 3:**
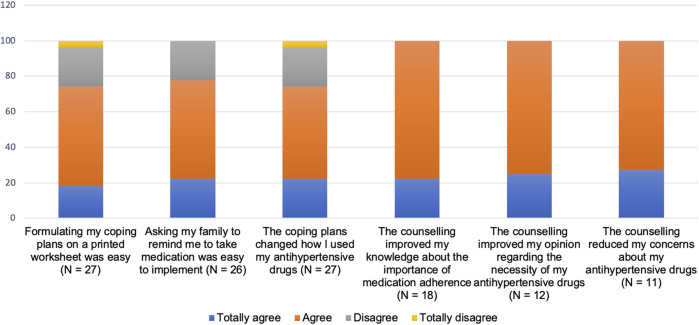
Patients’ experiences with implementing each component of the intervention based on barrier(s) for adherence identified.

On average, the duration of counselling lasted for 14.2 min (range 5–20 min) and 10.6 min (range 3–18 min) at baseline and 1-month follow-up session, respectively. Most patients (84%) reported that the duration of counselling was sufficient. Only three patients expressed that the counseling was too short, whereas four patients said it was too long (Table 2). Most patients receiving strategies addressing forgetfulness (totally) agreed that formulating coping plans and asking support from family member was easy to implement. For those receiving tailored counselling, all patients (totally) agreed that this counselling improved their knowledge about the importance of medication adherence, improved their opinion regarding the necessity of antihypertensive drugs and/or reduced their concerns about their antihypertensive drugs (Figure 3).

### Maintenance

All pharmacists mentioned that the intervention can be maintained when the number of pharmacists in the CHC is sufficient regarding required time investment for the intervention. Furthermore, they would need support from health care authorities, that is, the head of the CHCs and the Department of Health of Bandung City, Indonesia, for conducting such a service. Particularly, the pharmacists wanted clear and practical guidelines on medication adherence counselling. All pharmacists were willing to continue the intervention as part of routine clinical practice in the future. Four of them also wanted to use the intervention for other diseases. When the intervention would be implemented in the future, all but one of the patients would like to receive the counselling either at every regular visit or at least once per six months (Table 2).

## Discussion

The tailored pharmacist-led medication adherence intervention in patients with diabetes was well received and implemented in the participating Indonesian CHCs. It showed high participation rates and generally satisfied both pharmacists and patients. The pharmacists were able to identify individual patient adherence barriers and were easily guided to the corresponding adherence strategies using the support tools. In most cases, the elements of the intervention were carried out by pharmacists as intended, and the patients reported that the tailored intervention improved their knowledge and opinion about antihypertensive drugs. The training of pharmacists as well as having practical support tools, including a simple flow-chart, an adherence intervention wheel, and a personalized patient leaflet, seemed to have facilitated the implementation of the intervention in a routine care setting. All pharmacists were willing to continue the intervention as part of routine clinical practice in the future, and most patients would like to receive the counselling regularly. Some suggestions were made by the pharmacists for strengthening the intervention, such as having sufficient time, including home visits for specific patients, involving other health care providers, and availability of a personal counseling room. Furthermore, practical guidelines on medication adherence counseling and support of health care authorities would be helpful to maintain this service.

A systematic review in low- and middle-income Asian settings showed that pharmacists’ knowledge, tools and skills may not be sufficient to maintain pharmacist-led interventions in the long-term (Miller and Goodman, 2016). As mentioned by the pharmacists in our study, more explicit guidelines for pharmaceutical care may be important for maintaining the adherence intervention. Furthermore, lack of time is a common barrier to the provision of medication adherence support by community pharmacists (Fénélon-Dimanche et al., 2020). Therefore, offering a short training and practical tools that support pharmacists to quickly identify non-adherent patients and target only non-adherent patients using a tailored approach may be important elements of our intervention. Challenges can be, however, to provide the pharmacists with sufficient time and their own counselling room to conduct the targeted and tailored intervention. This may require support from authorities both at the level of the CHC as at a national level. In Indonesia, the national guidelines for pharmaceutical care state that pharmacists need to emphasize the importance of medication adherence during patient visits to the CHC (Kementerian Kesehatan Republik Indonesia, 2016). However, they lack explicit guidance on when and how to identify and improve medication non-adherence during patient counselling. In European countries, there are more comprehensive guidelines and resolutions on the implementation of pharmaceutical care including the role of the pharmacist to improve medication adherence by patient counselling (Committee of Ministers Council of Europe, 2020). This can be explained by the fact that pharmacist-led interventions have shown to improve medication adherence (Presley et al., 2019; Reeves et al., 2020). For some patients, home visits may be needed in order to monitor medication adherence more objectively, especially for patients at risk of medication related problems. In our study, several patients were lost to follow-up because they did not visit the CHCs to refill their medication. These patients are likely to have more adherence problems as compared to those who did visit the CHCs in time to refill their medication. Recognition and interventions to address adherence barriers observed during home visit may improve medication adherence (Williams et al., 2006; Renfro et al., 2019).

From the patient perspective, it seems the intervention was much appreciated and helpful. It became clear that a tailored intervention fulfilled a need, since a substantial number of patients had problems with forgetting their medication or had insufficient knowledge or insufficiently addressed concerns regarding their antihypertensive drugs. The high satisfaction reported by patients is important, since patients who are satisfied with pharmacy services are more likely to adhere to their medication (Gu et al., 2008). Our findings suggest that there are opportunities for scaling up the intervention to larger populations in Indonesia. Future research should focus on the optimal frequency of regular counselling since developing new habits may take up to one year (Lally et al., 2010).

This process evaluation also provides insights on specific effects of the tailored intervention. Previously, we found that the intervention significantly improved patients’ adherence, but not their beliefs about medication (Alfian et al., 2021). Overall, adequate levels of reach, adoption and implementation of the intervention explains its effect on adherence. The lack of an overall effect on beliefs about medication can be explained by the fact that our intervention was tailored to needs of the patients, and focused more on forgetfulness than on lack of knowledge or motivation. The patients with a lack of motivation did report that the counselling improved their opinion regarding the necessity of antihypertensive and reduced their concerns but it seemed that lack of motivation was more persistent. Changing lack of motivation and underlying negative beliefs about medication may require more than two sessions of counselling by a pharmacist (Zwikker et al., 2014; Scala et al., 2018). It might be that involvement of other health care providers, as suggested by one of the pharmacists in our study, could be of help.

A strength of this study is that we used the RE-AIM framework to systematically evaluate the implementation of the intervention. The advantage of the RE-AIM framework is that it captures both quantitative and qualitative measures of contextual factors (Farris et al., 2007). Furthermore, both pharmacists and patients’ perspectives were explored, allowing us to have a comprehensive evaluation during the implementation of the intervention. All pharmacists that were asked to participate in the intervention study were willing to do so. These were pharmacists on duty in CHCs with the highest number of diabetes patients in Bandung City, Indonesia. Some limitations need to be mentioned. The focus group discussion only included the five pharmacists randomized to the intervention who may not be fully representative of other pharmacists in Bandung City or in Indonesia. A larger group might have generated more views on the further implementation of the intervention. Furthermore, some of the included patients were lost to follow-up. These could in part be patients that were less interested in the intervention or had more difficulties with being adherent. Therefore, our included patients might be more willing to change their non-adherent behavior to antihypertensive drugs and be more satisfied with the intervention. Regarding their sociodemographic characteristics, however, the patients with one or two sessions appeared to be similar. We do not know to what extent our included patients were representative of all patients with diabetes and hypertension in Bandung City or in Indonesia but we included patients with a broad range in demographic characteristics. Importantly, we included both younger and older patients, patients with limited education and patients with different types of health insurance. Finally, the implementation of the different aspects of the intervention was measured using a pharmacist self-reported checklist and a patient questionnaire, which may lead to more socially desirable answers. Audio and/or video recordings would have allowed for a more objective assessment of the counselling.

## Conclusion

The positive effects of the pharmacist-led intervention can be explained by adequate levels of reach, adoption and implementation in the setting of the participating CHCs. Tailoring the support strategies to the patients’ needs and using practical support tools resulted in positive responses from both pharmacists and patients. Scaling up the intervention to larger populations in Indonesia and other low- and middle-income countries may require additional actions. Relevant factors to consider include additional training of pharmacists in motivational counselling, sufficient time, home visits for specific patients, involving other health care providers to improve medication adherence and pharmaceutical care, providing pharmacists with their own counselling room, explicit guidance for pharmacists on medication adherence counselling, and support from health care authorities.

## Data Availability

The raw data supporting the conclusions of this article will be made available by the authors, without undue reservation.
