# Supplementary material for: Process Evaluation of Implementing a Pharmacist-Led Intervention to Improve Adherence to Antihypertensive Drugs Among Patients with Type 2 Diabetes in Indonesian Community Health Centers
Source: Front Pharmacol. 2021 May 5;12:652018. doi: 10.3389/fphar.2021.652018 (PMC8131821; doi:10.3389/fphar.2021.652018)
Supplement: Supplementary file 1 [file DataSheet1.docx]

**Process evaluation of implementing a pharmacist-led intervention to improve adherence to antihypertensive drugs among patients with type 2 diabetes in Indonesian Community Health Centers**

Sofa D. Alfian, Job F. M. van Boven, Aulia Iskandarsyah, Rizky Abdulah, Eelko Hak, Petra Denig

**SUPPLEMENTARY MATERIALS**

Table S1. Outline and list of questions of focus group discussion to evaluate pharmacists’ adoption, implementation, and maintenance of the intervention

| Opening | 1. Welcoming 2. Briefly introduce moderator and pharmacists 3. Explanation about the topics that will be discussed 4. Explanation that there are no right or wrong answers, only personal opinions and points of view (moderator will guide the discussion). Explain that the discussion will be recorded, transcribed and analysed anonymously. They have to sign informed consent for this (distribute forms to sign at this point). |
| --- | --- |
| List of questions for each dimension: | |
| Adoption | What do you think of the program in general? (opening question that all should answer) (Number of sessions, time between sessions, duration of sessions) |
|  | What do you think of each component of the intervention?  (Communication training, habit-based strategies, family support, counselling to educate and to motivate patients and other drug related problems). What do others think? |
|  | Tell me about positive experiences you have had with the program. Do others have agreed? Do others have different experiences? |
|  | Tell me about negative experiences you have had with the program. Do others agree? Do others have different experiences? |
| Implementation | Suppose that you were in charge and could make one change that would make the program better. What would you do? What do the others think? |
|  | Suppose that you were in charge, do you think this program could be implemented in your CHC? Please explain why or why not. What do others think? What do you see as barriers? What would help to support the implementation? |
| Maintenance | Suppose that this program is implemented, do you think this program will sustain in your CHC? Please explain why or why not. What do others think? What do you see as barriers? What would help to sustain this program? |
| Closing | 1. Is there anything you would like to add regarding this program that has not yet been addressed? (Do a round, so that all can give their final comments) 2. Thank people and tell them what we will do with the results (and how they will be informed about the results when they want that). |

Table S2. Survey about patient satisfaction with the program

| In the past months, you received a newly developed pharmacy service to support the optimal use of your antihypertensive drugs. We hope you will fill out this survey to tell us about your experience with this service and any ways it can be improved. For each of the statements, please tick one answer, which best applies to you. | | | | |
| --- | --- | --- | --- | --- |
| **Evaluation of pharmacy service in general:** | | | | |
| 1. How satisfied are you with the information provided by the pharmacist regarding your antihypertensive drugs during the past three months? | | | | |
| Very unsatisfied | Unsatisfied | Neutral | Satisfied | Very satisfied |
| 2. How important do you think it is that the pharmacist provides information about your antihypertensive drugs? | | | | |
| Very unimportant | Unimportant | Neutral | Important | Very important |
| 3. Suppose the service will be implemented in the future, how often would you like to receive it? | | | | |
| At every visit | Once per 6 months | Once per year | Once per 2 years | Never |
| **Evaluation of the implementation of each component of the program:** | | | | |
| 1. Coping plans | | | | |
| 1. Was it easy to formulate a coping plan on a printed worksheet? | | | | |
| Totally agree | Agree | Disagree | Totally disagree | Not applicable |
| 1. The coping plans changed how I acted toward my antihypertensive drugs | | | | |
| Totally agree | Agree | Disagree | Totally disagree | Not applicable |
| 2. Family support | | | | |
| Asking my family to remind me to take medication was easy to implement | | | | |
| Totally agree | Agree | Disagree | Totally disagree | Not applicable |
| Pharmacist counseling | | | | |
| 1. Information provided by the pharmacist was clear | | | | |
| Totally agree | Agree | Disagree | Totally disagree |  |
| b. The counseling improved my knowledge about the importance of medication adherence | | | | |
| Totally agree | Agree | Disagree | Totally disagree |  |
| c. I had sufficient opportunity to discuss my own experiences and problems with my medication | | | | |
| Totally agree | Agree | Disagree | Totally disagree |  |
| d. The pharmacist listened well to my own experiences and problems with medication | | | | |
| Totally agree | Agree | Disagree | Totally disagree |  |
| e. The counseling improved my opinion regarding the necessity of my antihypertensive drugs | | | | |
| Totally agree | Agree | Disagree | Totally disagree |  |
| f. The counseling reduced my concerns about my antihypertensive drugs | | | | |
| Totally agree | Agree | Disagree | Totally disagree |  |
| g. The counseling addressed other drug related problems I had | | | | |
| Totally agree | Agree | Disagree | Totally disagree |  |
| **Please rate your satisfaction about the general visits characteristics:** | | | | |
| 1. General atmosphere during the sessions | | | | |
| Very tensed | Tensed | Relaxed | Very relaxed |  |
| 2. Number of sessions | | | | |
|  | Too less | Sufficient | Too much |  |
| 3. Time between sessions (1 month) | | | | |
|  | Too short | Sufficient | Too long |  |
| 4. Duration of sessions | | | | |
|  | Too short | Sufficient | Too much |  |
| How could we improve the program? | | | | |

Table S3. Checklist for pharmacist to ensure intervention fidelity

Please tick the box which best applies to you for each of the items you have done at each patient visit

| **Baseline (T0) : Date : DD-MM-YY** | **Not at all** | **Minimally** | **To some extent** | **A good deal** | **A great extent** |
| --- | --- | --- | --- | --- | --- |
| 1. Reminder/habit-based strategies/family member support |  |  |  |  |  |
| 1. Counselling to increase patients’ knowledge using teach-back method |  |  |  |  |  |
| 1. Counselling to increase patients’ motivation |  |  |  |  |  |
| 1. Explore/address other drug related problems |  |  |  |  |  |
| I confirm that the data are complete and accurate.  Pharmacist signature:  Pharmacist printed name: | | | | | |
| **Follow-up assessment (T1): Date: DD-MM-YY** |  |  |  |  |  |
| Review and discuss patient’s implementation and experiences with the discussed plans and recommendations. |  |  |  |  |  |
| Identify any possible barriers that were not yet addressed during the first session: |  |  |  |  |  |
| 1. Reminder/habit-based strategies/family member support |  |  |  |  |  |
| 1. Counselling to increase patients’ knowledge using teach-back method |  |  |  |  |  |
| 1. Counselling to increase patients’ motivation |  |  |  |  |  |
| 1. Explore/address other drug related problems |  |  |  |  |  |
| I confirm that the data are complete and accurate.  Pharmacist signature:  Pharmacist printed name: | | | | | |

Note: Scoring: “not at all”: 0; “minimally”: 1; “to some extent”: 2; “a good deal”: 3; “a great extent”: 4
